# The Interaction between Circulating Complement Proteins and Cutaneous Microvascular Endothelial Cells in the Development of Childhood Henoch-Schönlein Purpura

**DOI:** 10.1371/journal.pone.0120411

**Published:** 2015-03-11

**Authors:** Yao-Hsu Yang, I-Jung Tsai, Chun-Jung Chang, Ya-Hui Chuang, Hui-Yao Hsu, Bor-Luen Chiang

**Affiliations:** 1 Department of Pediatrics, National Taiwan University Hospital, College of Medicine, National Taiwan University, Taipei, Taiwan; 2 Graduate Institute of Oncology, College of Medicine, National Taiwan University, Taipei, Taiwan; 3 Department of Clinical Laboratory Sciences and Medical Biotechnology, College of Medicine, National Taiwan University, Taipei, Taiwan; 4 Department of Medical Research, National Taiwan University Hospital, Taipei, Taiwan; 5 Graduate Institute of Clinical Medicine, College of Medicine, National Taiwan University, Taipei, Taiwan

## Abstract

**Objective:**

In addition to IgA, the deposition of complement (C)3 in dermal vessels is commonly found in Henoch-Schönlein purpura (HSP). The aim of this study is to elucidate the role of circulating complement proteins in the pathogenesis of childhood HSP.

**Methods:**

Plasma levels of C3a, C4a, C5a, and Bb in 30 HSP patients and 30 healthy controls were detected by enzyme-linked immunosorbent assay (ELISA). The expression of C3a receptor (C3aR), C5a receptor (CD88), E-selectin, intercellular adhesion molecule 1 (ICAM-1), C3, C5, interleukin (IL)-8, monocyte chemotactic protein (MCP)-1, and RANTES by human dermal microvascular endothelial cells (HMVEC-d) was evaluated either by flow cytometry or by ELISA.

**Results:**

At the acute stage, HSP patients had higher plasma levels of C3a (359.5 ± 115.3 vs. 183.3 ± 94.1 ng/ml, *p* < 0.0001), C5a (181.4 ± 86.1 vs. 33.7 ± 26.3 ng/ml, *p* < 0.0001), and Bb (3.7 ± 2.6 vs. 1.0 ± 0.6 μg/ml, *p* < 0.0001), but not C4a than healthy controls. Although HSP patient-derived acute phase plasma did not alter the presentation of C3aR and CD88 on HMVEC-d, it enhanced the production of endothelial C3 and C5. Moreover, C5a was shown *in vitro* to up-regulate the expression of IL-8, MCP-1, E-selectin, and ICAM-1 by HMVEC-d with a dose-dependent manner.

**Conclusion:**

In HSP, the activation of the complement system in part through the alternative pathway may have resulted in increased plasma levels of C3a and C5a, which, especially C5a, may play a role in the disease pathogenesis by activating endothelium of cutaneous small vessels.

## Introduction

Henoch-Schönlein purpura (HSP) primarily affects children with an annual incidence of 13–20 cases per 100 000 < 17 years old children [1,2]. It is a kind of leukocytoclastic vasculitis involving small vessels and clinically characterized by non-thrombocytopenic cutaneous palpable purpura, arthritis/arthralgia, bowel angina, gastrointestinal bleeding, and hematuria/proteinuria. Most cases of HSP occur in autumn and winter and are often preceded by an upper respiratory tract infection [3,4]. In addition, some medicines and vaccines had been reported to be associated with HSP [5,6,7,8,9,10]. Together with the deposition of IgA on small vessel wall and the infiltration of polymorphonuclear neutrophils (PMN) around the vessel and in the vessel walls, HSP is now considered as an immune-mediated vasculitis possibly triggered by multiple environmental factors. However, the real pathogenesis is still unknown.

The significance of adaptive immunity in HSP has been extensively studied [4,11,12,13,14]. Increased type 3 T helper (TH3) cells and TH17 cells were identified at the acute stage of HSP [11,12]. Recently, we have found that HSP-derived IgA bound well to β2 glycoprotein I (β2GPI) and several fragmental peptides of β2GPI [13]. These β2GPI-reactive IgA then crossly reacted with endothelial cells (EC) to induce interleukin (IL)-8 production by EC through the MEK/ERK signaling pathway [14]. In the presence of complement, the above IgA could damage EC in vitro [13]. Besides, the deposits of complement (C)3 are often shown on patients’ skin and renal biopsies. Combined, in addition to adaptive immunity, innate immunity like complement is likely to play a role in the development of HSP.

The complement system consisting of a number of small proteins is an important component of host defense. There are three different pathways of complement activation: the classical pathway, the lectin pathway, and the alternative pathway. The classical pathway is initiated mainly by antigen-antibody complexes; whereas mannan-binding lectin binding to pathogen surfaces triggers the lectin pathway. In alternative pathway, C3 undergoes spontaneous hydrolysis on many microbial surfaces [15]. However, in addition to above major initiators, the complement system can also be activated by other fctors. Of them, IgA has been shown to activate both lectin and alternative pathways [16,17]. Moreover, it is noticeable that some circulating bioactive proteins (e.g. C3a and C5a) generated in the process of complement activation have been found pathogenically relevant in various inflammatory disorders [18,19].

Accordingly, it is hypothesized that the circulating complement proteins are involved and play a pathogenic role in HSP. In this study, we first measured serum or plasma levels of various complement proteins to test the hypothesis. The contribution of such activation to the pathogenesis of HSP was then studied, especially focusing on the interaction between C3 (C3a)/C5 (C5a) and EC. We would evaluate the expression of some chemokines and adhesion molecules by C3a/C5a activated-EC, which are critically involved in the recruitment of PMN.

## Materials and Methods

### Patients and controls

Based on the updated HSP diagnostic criteria and classification defined by the European League Against Rheumatism, Paediatric Rheumatology International Trials Organisation, and Paediatric Rheumatology European Society (EULAR/PRINTO/PRES) 2010 [20,21], those children with acute HSP were included in this study. Their clinical presentations were recorded; blood samples were collected at the acute stage before steroid or other immunosuppressants treatment and at the convalescent stage, which is defined as discontinuation of medications and without any symptoms and signs of HSP. Some age- and sex-matched healthy children were recruited as controls. The written informed consents were obtained from both children and their guardians, and this study had been approved by the Research Ethic Committee of National Taiwan University Hospital (NTUH).

### Serum C3 and C4 detection

The serum levels of C3 and C4 in HSP patients at both acute and convalescent stages were determined at the central laboratory of NTUH by nephelometry.

### Detection of complement proteins and chemokines by enzyme-linked immunosorbent assay (ELISA)

The plasma levels of C3a, C5a, C4a, Bb of HSP patients and healthy controls, the levels of C3 and C5 in culture supernatants, and the levels of IL-8, monocyte chemotactic protein (MCP)-1, and RANTES in culture supernatants and plasma of patients and controls were detected according to the manufacturer’s instructions of commercial ELISA kits. Human C3 and C5 ELISA kits were purchased from Assaypro (Assaypro, St. Charles, MO, USA). Human C3a, C4a, and C5a ELISA kits were purchased from BD Biosciences (BD Biosciences, Franklin Lakes, NJ, USA), Bb from Quidel (Quidel Corporation, San Diego, CA, USA). Human IL-8, MCP-1, and RANTES ELISA kits were purchased from R&D Systems (R&D Systems, Minneapolis, MN, USA).

### C3a receptor (C3aR) and C5a receptor (CD88) expression by EC

The heterogeneity of biologic properties in EC of different origins (e.g. EC derived from large/medium/small vessels or from different organs/tissues) has been well characterized [22]. Since HSP is small vessels vasculitis that occurs mostly in cutaneous capillaries and postcapillary venules [3,4], we used primary human dermal microvascular endothelial cells (HMVEC-d, Lonza, Walkersville, MD, USA) as the targets for subsequent experiments. Of note, all studies were performed on cells between passages 3 and 11. HMVEC-d were seeded on gelatin-coated 12-well plates at a density of 5×10^4^ cells/well. When the cellular growth became confluent, plasma of patients with acute HSP, plasma of healthy controls, or complete culture medium (EGM-2MV, Clonetics) alone (100 μl) were added to each well to co-culture with HMVEC-d for further 48 hr. Cells were then harvested by trypsin, washed by PBS, and labeled by PE-conjugated mouse IgG anti-human C3aR, IgG anti-human CD88, or IgG isotype control (BD Biosciences) at 4°C for 30 min. Stained cells were assayed by a FACSCalibur using the CellQuest Software (BD Biosciences).

### C3 and C5 expression by EC

HMVEC-d at a density of 1×10^4^ cells/well were seeded on gelatin-coated 96-well microtitre plates (Nunc, Demark) and incubated in complete culture medium for 3–4 days. When the cellular growth became confluent, they were treated with plasma of patients with acute HSP, plasma of healthy controls, and medium alone (40 μl), respectively at 37°C for 48 hr. Subsequently, the culture medium with/without human plasma was discarded and the cells were washed by PBS. HMVEC-d were then incubated with serum free medium at 37°C. Forty-eight hours later, supernatants were collected for C3 and C5 detection by commercial ELISA kits.

### Chemokines production by C3a/C5a-treated EC

HMVEC-d (1×10^4^ cells/well) were seeded on gelatin-coated 96-well microtitre plates and incubated in complete culture medium till the cellular growth became confluent. Before the treatment of cells, culture medium was removed from each well which was then washed by PBS. The native human C3a (Calbiochem, Merck KGaA, Darmstadt, Germany) or recombinant human C5a (BioVision, Milpitas, LA, USA) of different concentrations (0, 25, 50, 100, 200 ng/ml) was added to wells containing HMVEC-d and serum free medium. Twenty-four hours later, the supernatants were collected and the levels of IL-8, MCP-1, and RANTES were assayed by commercial ELISA kits.

### Adhesion molecules expression on C3a/C5a-treated EC

HMVEC-d at a density of 5×10^4^ cells/well were seeded on gelatin-coated 12-well plates with complete culture medium for 3–4 days. C3a, C5a of different concentrations (0, 50, 100, 200 ng/ml), or tumor necrosis factor α (TNF-α, 10 ng/ml) were then added in each well. Following incubation for 6 hr (E-selectin analysis) or 18 hr (intercellular adhesion molecule 1, ICAM-1 analysis), the cells were detached from plates and washed. The cell suspension was centrifuged, the supernatant was discarded and the pellet was re-suspended. These cells were then stained by adding APC-conjugated mouse anti-human E-selectin monoclonal antibody (BD Biosciences) or PE-conjugated mouse anti-human ICAM-1 (CD54) monoclonal antibody (Serotec, USA) at 4°C for one hr. Isotype matched control mouse IgG was used to eliminate non-specific bindings. The final results were analyzed using FACSCalibur.

### Statistical analysis

The expression levels of C3aR, CD88, E-selectin, and ICAM-1 were presented as mean fluorescence intensity (MFI). The values of all variables were expressed as means ± SD. Each two-group comparison was conducted using Student’s *t*-test except that paired *t*-test was used to estimate the differences of C3, C4, C3a, and C5a between acute and convalescent stages in HSP patients. A two-tailed *p* value of less than 0.05 was considered statistically significant.

## Results

### Clinical characteristics

Thirty children (15 males and 15 females) with HSP were recruited in this study from Jan 2010 to Dec 2013. The average ages (in years) at the diagnosis (acute stage) were 7.3 with a range from 2.6 to 17. Three children were younger than 4 years of age (2 males and 1 female) and 9 children were older than 8 years (4 males and 5 females). In addition, another 30 age- and sex-matched healthy children were enrolled as controls. All HSP patients (100%) presented with skin purpura, especially on lower extremities and buttocks. Arthralgia and/or arthritis occurred in 17 patients (56.7%), and the most commonly affected joints were ankles and knees. Abdominal pain was noted in 16 patients (53.3%) and 5 of them were positive for stool occult blood test. Renal involvement presenting as hematuria and/or proteinuria was found in 6 patients (20%). Although the disease courses were varied, they all recovered completely within 2 months. The average follow-up duration was 4 months.

### Serum levels of C3 and C4 in HSP patients

**Fig 1:**
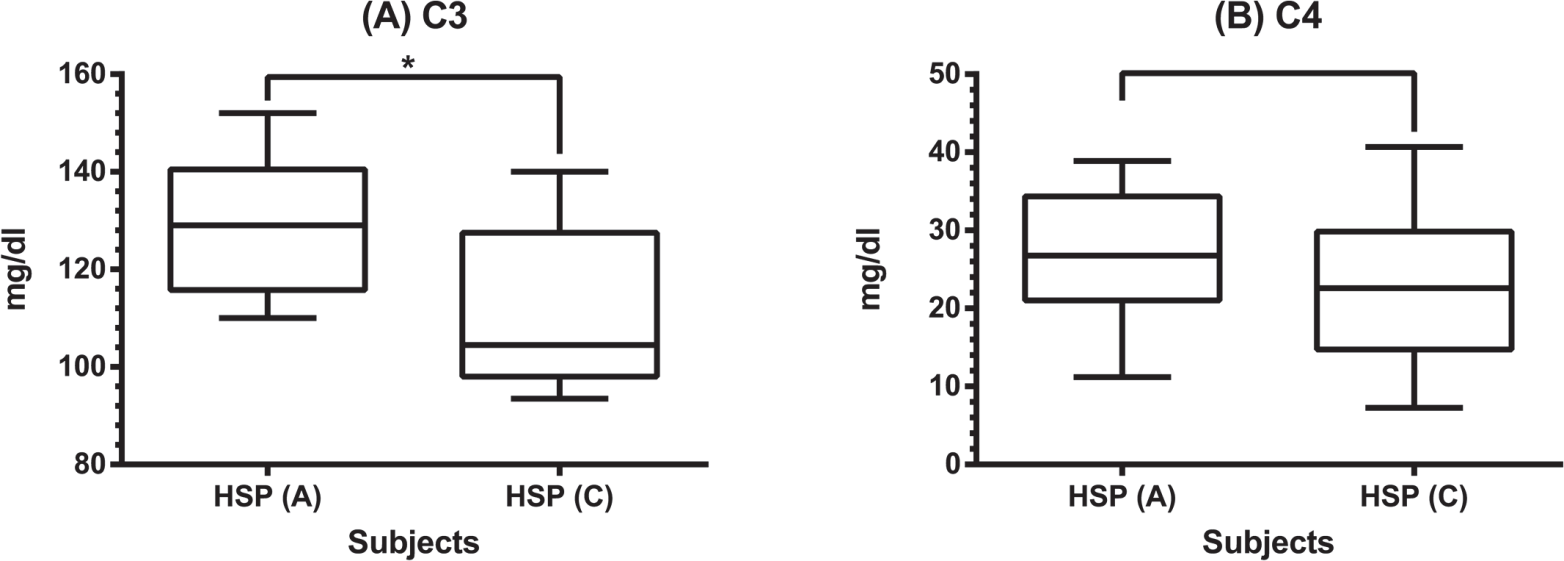
The serum levels of C3 and C4 in 30 HSP patients. Serum C3 (A) and C4 (B) levels were detected by nephelometry in HSP patients between acute and convalescent stages. * denotes *p* < 0.001.

To clarify the activation of complement in HSP, we first evaluated the serum levels of C3 and C4 in patients at both acute and convalescent stages. The results showed that C3 and C4 serum levels in all patients, no matter at acute stage or at convalescent stage, were within normal ranges (reference values, C3: 90–180 mg/dl, C4: 10–40 mg/dl). However, C3 (Fig. 1A) but not C4 (Fig. 1B) serum levels were significantly decreased when the disease subsided (C3: 128.8 ± 14.3 *vs* 111.8 ± 17.0 mg/dl, *p* < 0.001; C4: 26.6 ± 8.3 vs 22.8 ± 10.1 mg/dl, *p* = 0.1).

### Plasma levels of circulating complement fragments in HSP patients

**Fig 2:**
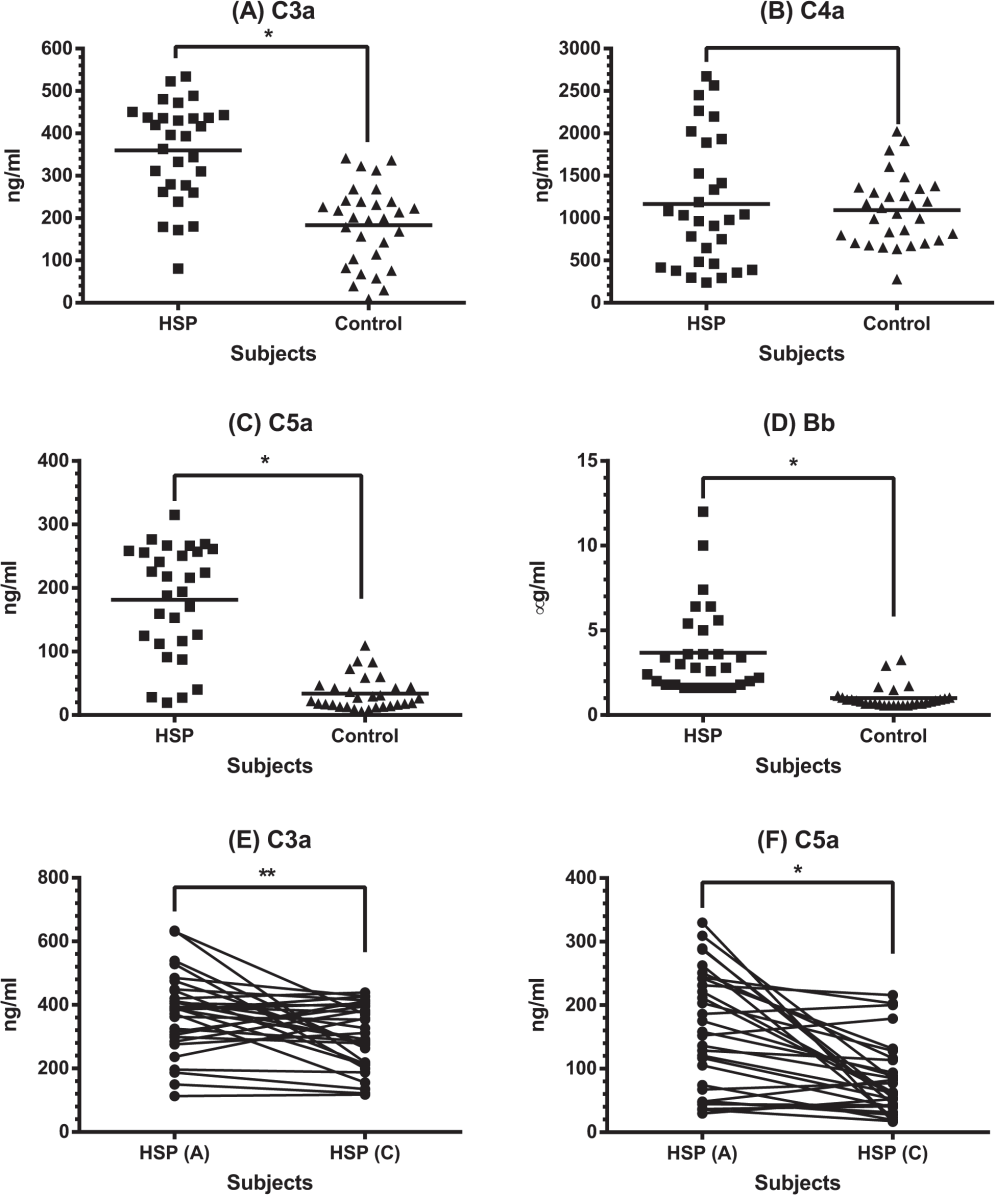
The plasma levels of complement proteins in HSP patients. Plasma C3a (A), C4a (B), C5a (C), and Bb (D) levels were determined by ELISA between 30 patients with acute HSP and 30 healthy controls. * denotes *p* < 0.0001. Plasma C3a (E) and C5a (F) levels in HSP patients between acute and convalescent stages were compared. * denotes *p* < 0.0001, ** denotes *p* = 0.007.

In addition to C3, C4, we further evaluated the plasma levels of some circulating complement fragments including C3a, C4a, C5a, and Bb in 30 children with acute HSP and 30 healthy controls. As can be seen in Fig. 2A-D, patients with acute HSP had higher plasma levels of C3a (359.5 ± 115.3 *vs* 183.3 ± 94.1 ng/ml, *p* < 0.0001), C5a (181.4 ± 86.1 *vs* 33.7 ± 26.3 ng/ml, *p* < 0.0001), and Bb (3.7 ± 2.6 *vs* 1.0 ± 0.6 μg/ml, *p* < 0.0001) than healthy controls, whereas there was no difference of C4a levels between patients and controls (1166.0 ± 760.8 *vs* 1093.0 ± 412.5ng/ml, *p* = 0.64). Since C3a and C5a have proinflammatory properties that may be related to disease activity, their plasma levels of each patient were measured at the convalescent stage. As shown in Fig. 2E and F, both C3a and C5a plasma levels declined significantly when compared with those in acute stage (C3a: 373.1 ± 126.2 *vs* 301.3 ± 100.2 ng/ml, *p* = 0.007; C5a: 159.7 ± 93.5 *vs* 81.4 ± 57.1 ng/ml, *p* < 0.0001).

### The effect of HSP plasma on C3aR and CD88 expression by HMVEC-d

**Fig 3:**
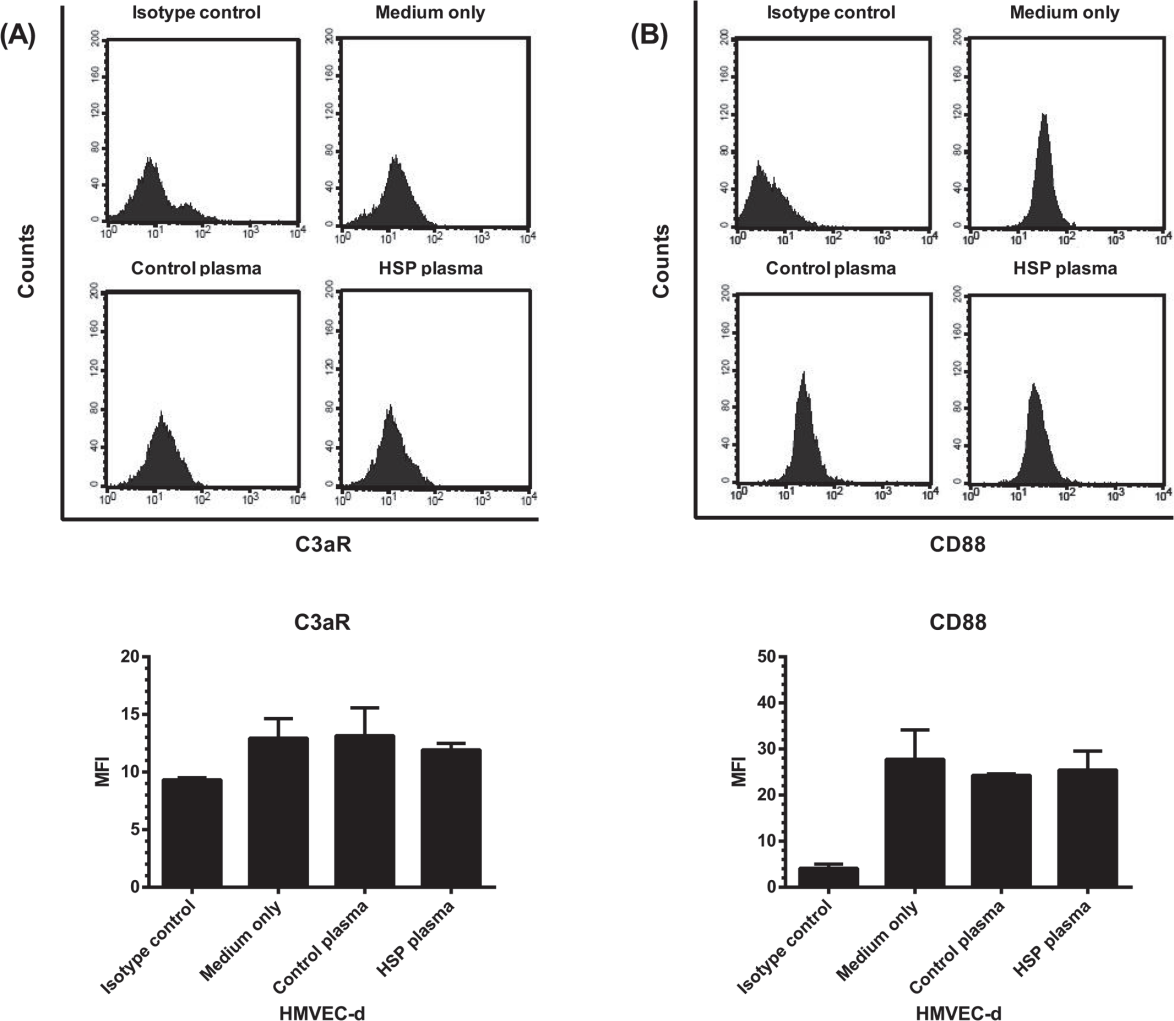
The expression of C3a receptor (C3aR) and C5a receptor (CD88) by HMVEC-d. HMVEC-d were pre-incubated with plasma of patients (N = 30) with acute HSP, plasma of healthy controls (N = 30), or culture medium alone for 48 hr, and then the cells were harvested and analyzed for the expression of C3aR (A) and CD88 (B) by flow cytometry. The expression levels were presented as mean fluorescence intensity (MFI).

The interaction between C3a/C5a and HMVEC-d was subsequently studied to explore the possible contribution of such bioactive complement proteins to HSP development. First, the expression of C3a and C5a receptors by HMVEC-d was analyzed. Fig. 3 showed that HMVEC-d constitutively expressed C3aR and CD88 (isotype control *vs* medium only). Moreover, the endothelial expression levels of both C3aR (Fig. 3A) and CD88 (Fig. 3B) were not significantly affected by the incubation with plasma of patients with acute HSP (HSP plasma vs control plasma, C3a: 11.9 ± 0.6 vs 13.2 ± 2.4, *p* = 0.56; C5a: 25.5 ± 4.1 *vs* 24.3 ± 0.3 ng/ml, *p* = 0.65).

### The effects of C3a and C5a on chemokines production by HMVEC-d

**Fig 4:**
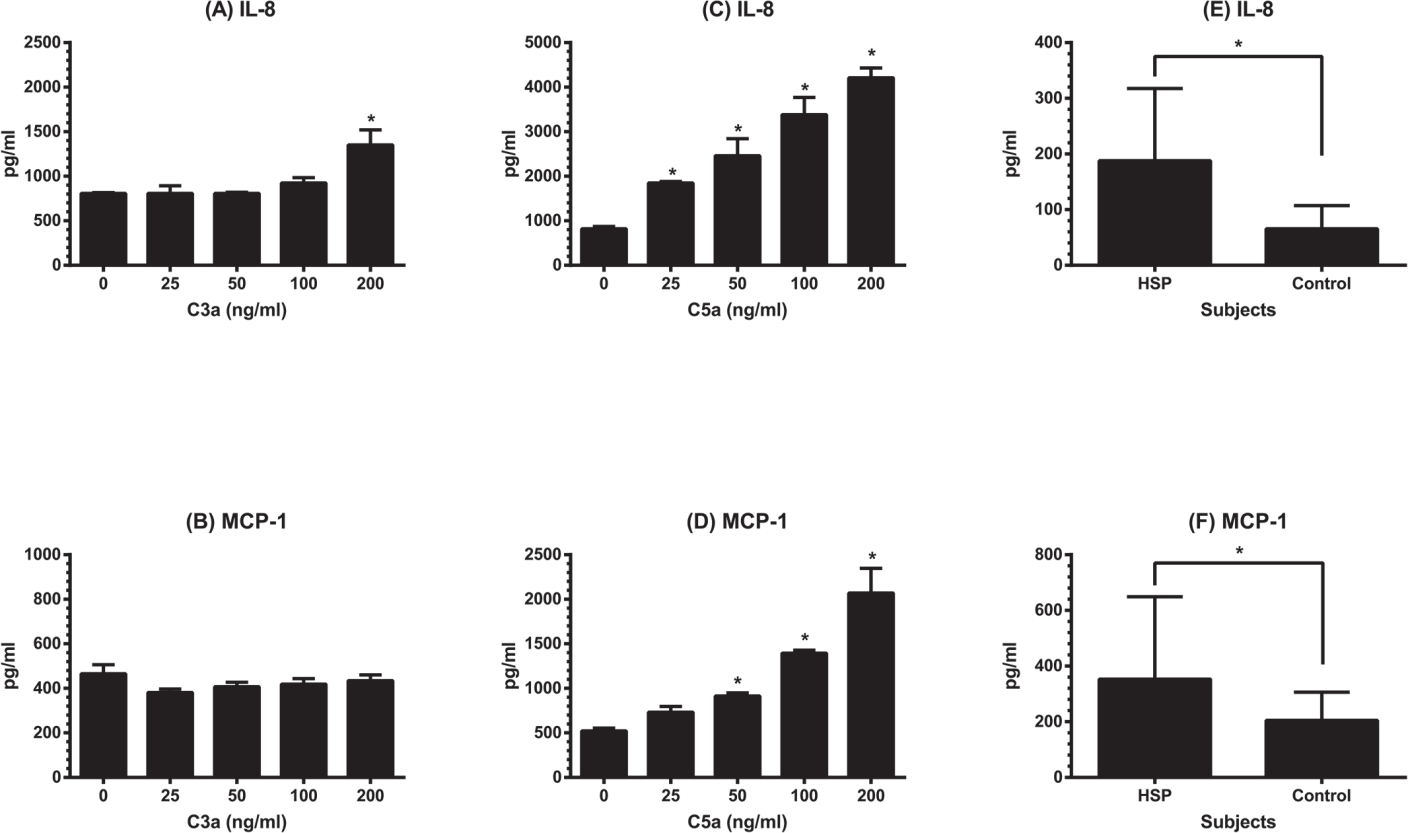
The production of endothelial IL-8 and MCP-1 and their plasma levels in HSP. HMVEC-d were first cultured in complete medium which contains fetal calf serum till their growth became confluent, and then incubated in serum free medium. C3a or C5a of different concentrations (0, 25, 50, 100, and 200 ng/ml) was added to each well for further 24 hr, the supernatant of each well was collected to test the levels of IL-8 secreted by C3a-treated HMVEC-d (A), MCP-1 secreted by C3a-treated HMVEC-d (B), IL-8 secreted by C5a-treated HMVEC-d (C), and MCP-1 secreted by C5a-treated HMVEC-d (D). * denotes *p* < 0.05, compared with the value of nontreated. Using the same ELISA kits, plasma levels of IL-8 (E) and MCP-1 (F) were evaluated in 30 patients with acute HSP and 30 healthy controls. * denotes *p* < 0.05.

Considering the results of increased plasma levels of C3a and C5a in children with acute HSP and the constitutive expression of C3R and CD88 on HMVEC-d, we further wanted to test whether C3a and C5a could induce the secretion of chemokines by HMVEC-d that assists in PMN accumulation and activation. After stimulating by C3a or C5a of different concentrations for 24 hr, the levels of IL-8, MCP-1, and RANTES in supernatants were analyzed. As can be seen in Fig. 4A-D, HMVEC-d secreted IL-8 and MCP-1 spontaneously in the culture medium. Under the treatment of C3a, endothelial IL-8 (Fig. 4A) and MCP-1 (Fig. 4B) levels were not changed except that C3a at a high concentration could significantly enhance the production of IL-8 by HMVEC-d (medium only *vs* C3a at 200 ng/ml: 806.5 ± 9.2 *vs* 1351.5 ± 169.1 pg/ml, *p* = 0.023). In contrast, C5a enhanced both IL-8 and MCP-1 production by HMVEC-d with a dose-dependent manner (Fig. 4C and D). On the other hand, RANTES was undetectable in culture supernatants of HMVEC-d with or without treatment by C3a and C5a. Of note, the original data were shown in Table 1. Thereafter, we evaluated the plasma levels of IL-8 and MCP-1 in patients with acute HSP. As shown in Fig. 4E and F, HSP patients at acute stage had higher IL-8 (187.7 ± 129.8 *vs* 65.4 ± 41.6 pg/ml, *p* < 0.001) as well as MCP-1 (353.3 ± 296.1 *vs* 204.8 ± 101.3 pg/ml, *p* = 0.013) levels than healthy controls.

**Table 1:** The expression of chemokines and adhesion molecules by C3a/C5a treated-HMVEC-d.

		Chemokine			Adhesion molecule	
		IL-8 (pg/ml)	MCP-1 (pg/ml)	RANTES (pg/ml)	E-selectin (MFI)	ICAM-1 (MFI)
C3a (ng/ml)	NT	806.5 ± 9.2	465.7 ± 40.5	ND	51.4 ± 9.9	101.5 ± 9.6
	25	806.5 ± 85.6	381.0 ± 16.0	ND		
	50	808.0 ± 11.3	407.3 ± 20.0	ND	51.2 ± 4.9	109.2 ± 11.7
	100	924.3 ± 59.2	419.0 ± 25.2	ND	57.8 ± 3.9	103.6 ± 16.6
	200	1351.5 ± 169.1*	434.3 ± 26.1	ND	57.5 ± 0.8	109.0 ± 20.1
C5a (ng/ml)	NT	819.0 ± 52.3	521.5 ± 31.8	ND	51.4 ± 9.9	101.5 ± 9.6
	25	1849.0 ± 34.0*	732.5 ± 64.3	ND		
	50	2461.0 ± 383.5*	913.5 ± 34.6*	ND	258.7 ± 45.2*	264.8 ± 7.8*
	100	3382.3 ± 388.3*	1394.5 ± 34.6*	ND	477.1 ± 25.2*	406.6 ± 6.0*
	200	4212.3 ± 217.9*	2069.0 ± 278.6*	ND	572.9 ± 98.3*	541.2 ± 91.4*

Abbreviations: NT, nontreated; ND, not detectable; IL-8, interleukin-8; MCP-1, monocyte chemotactic protein-1; ICAM-1, intercellular adhesion molecule-1; MFI, mean fluorescence intensity.

* *p* < 0.05, compared with the value of nontreated.

### The effects of C3a and C5a on adhesion molecules expression by HMVEC-d

**Fig 5:**
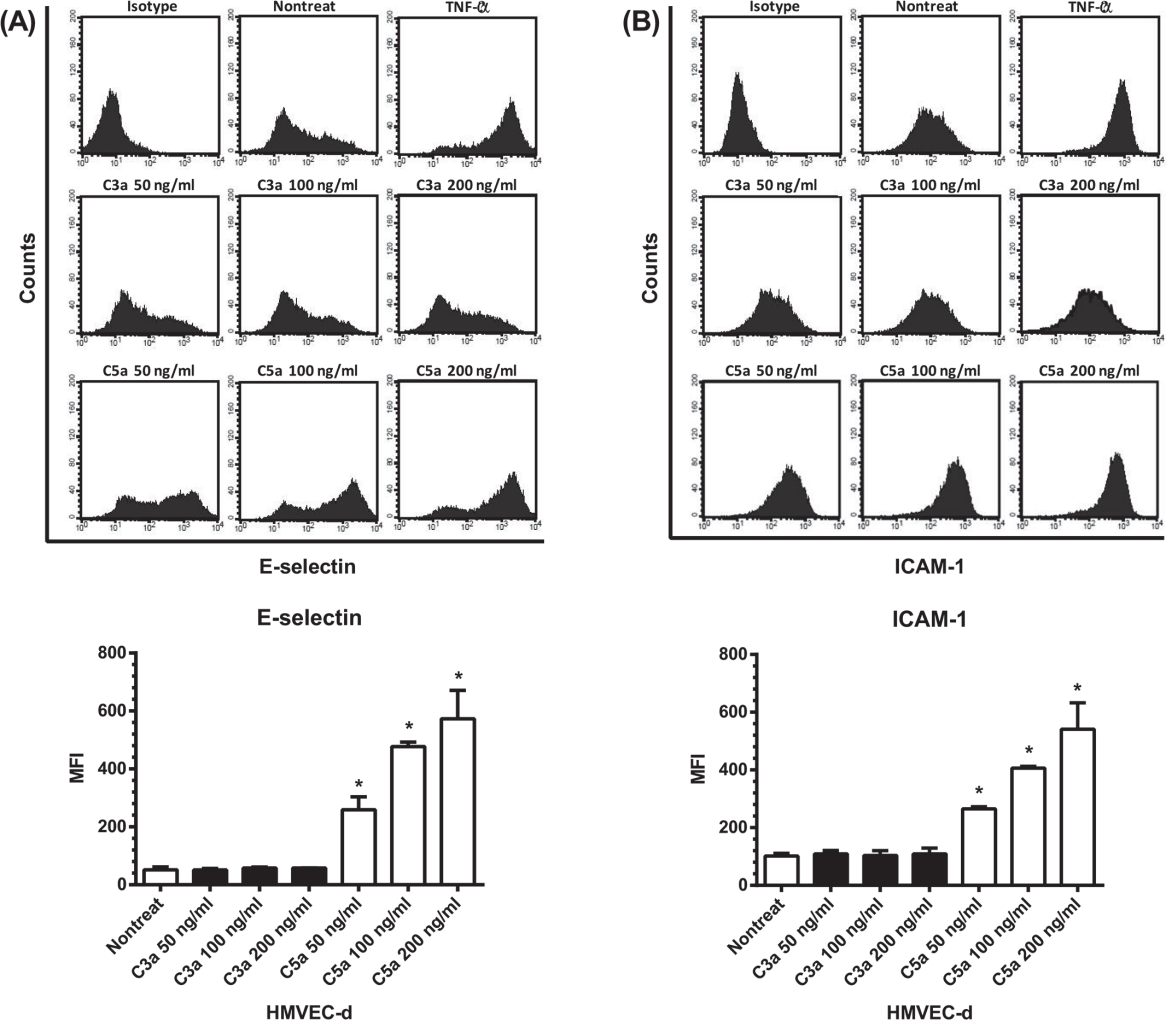
The expression of E-selectin and ICAM-1 by HMVEC-d. HMVEC-d were stimulated by C3a, C5a of different concentrations (0, 50, 100, 200 ng/ml), or TNF-α (10 ng/ml). Six hr and 18 hr later, the cells were harvested and analyzed for the expression of E-selectin (A) and ICAM-1 (B) by flow cytometry. The expression levels were presented as mean fluorescence intensity (MFI). * denotes *p* < 0.05, compared with the value of nontreated.

In addition to the effects on endothelial chemokines enhancement, we further evaluated the abilities of C3a and C5a to regulate the expression of adhesion molecules including E-selectin and ICAM-1 on HMVEC-d, that are critical molecules involving the PMN recruitment. As can be seen in Fig. 5, TNF-α which was used as a positive control had a powerful capacity to increase the expression of both E-selectin and ICAM-1 on HMVEC-d. C3a at different concentrations, however, did not alter the expression levels of E-selectin, whereas C5a could significantly enhance E-selectin expression on HMVEC-d with a dose-dependent relationship in this interaction (Fig. 5A). Similarly, there was a dose-dependent effect of C5a but not C3a on the enhancement of endothelial ICAM-1 expression (Fig. 5B). Of note, the original data were shown in Table 1.

### The effect of HSP plasma on C3 and C5 production by HMVEC-d

**Fig 6:**
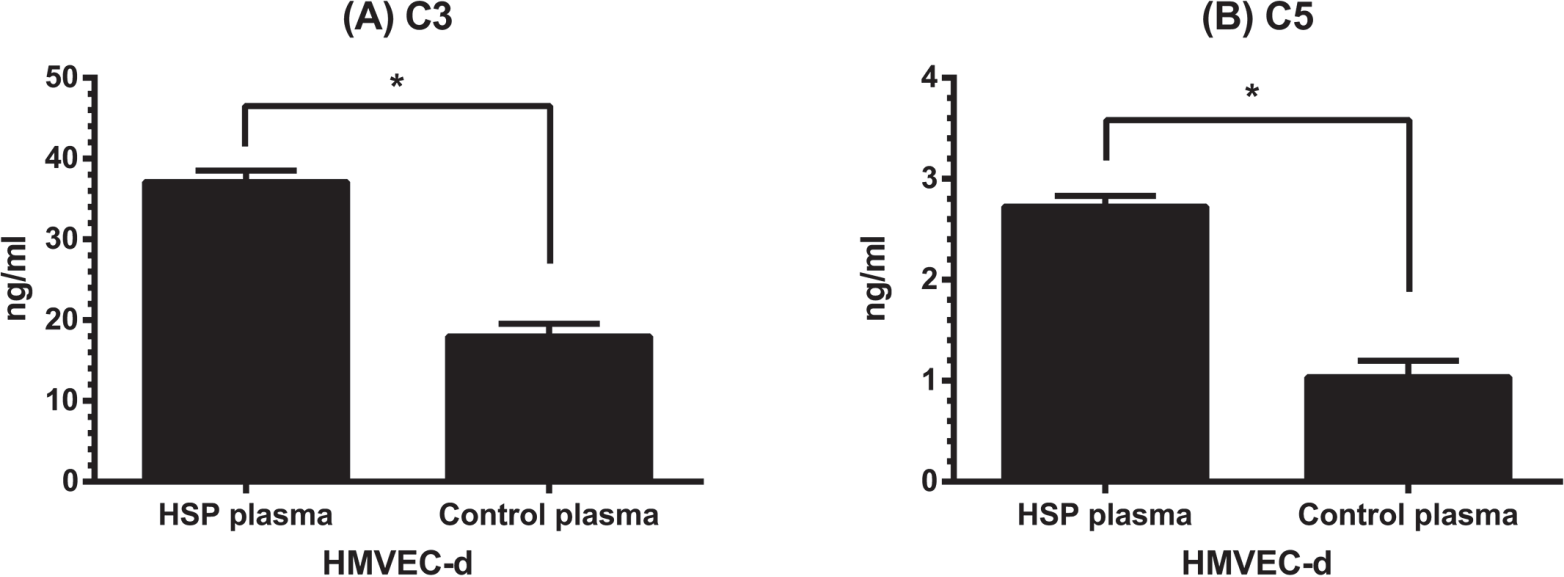
The production of C3 and C5 by HMVEC-d. HMVEC-d were pre-incubated with plasma of patients with acute HSP (N = 30) or plasma of healthy controls (N = 30) for 48 hr, and then the cells were washed and re-cultured in serum free medium for another 48 hr. C3 (A) and C5 (B) levels in culture supernatants were analyzed by ELISA. * denotes *p* < 0.01.

The potential of C3 and C5 production by HMVEC-d was also studied. The results showed that neither C3 nor C5 were detected by current ELISA kits in the culture supernatants (complete culture medium) of HMVEC-d. In contrast, for those HMVEC-d incubated with human plasma for 48 hr, they could later produce both C3 and C5 under the serum-free environment (Fig. 6). Moreover, HMVEC-d stimulated by plasma of patients with acute HSP produced more C3 (Fig. 6A) and C5 (Fig. 6B) than controls’ plasma-stimulated cells (C3: 37.1 ± 1.4 *vs* 18.0 ± 1.6 ng/ml, *p* = 0.006; C5: 2.7 ± 0.1 *vs* 1.0 ± 0.2 ng/ml, *p* = 0.007).

## Discussion

Immune complex-mediated diseases such as systemic lupus erythematosus frequently cause hypocomplementemia. HSP has also been regarded as an immune complex disease; however, hypocomplementemia with low serum or plasma C3 and/or C4 levels was not commonly shown and usually transient in patients with acute HSP [23,24]. In the present study, the results showed the serum levels of C3 and C4 in all 30 HSP children at both acute and convalescent stages were within normal ranges. Nevertheless, it is interesting to note that C3 but not C4 serum levels were significantly decreased at the convalescent stage. Combined with the pathological characteristic of C3 deposition on skin biopsies, it is conceivable that the complement activation is involved in the development of HSP.

We then evaluated which pathway was activated in these HSP children. By analyzing the plasma levels of certain complement fragments between HSP patients and healthy controls, we found that plasma C3a, C5a, and Bb levels were significantly higher in children with acute HSP than in controls, whereas there was no difference of C4a levels. Reviewing the complement cascade, C4 and C2 are activated in both classical and lectin pathways to generate a C3 convertase (C4b2a) and C4a. The hydrolyzed C3 in the alternative pathway binds to factor B resulting in the formation of a distinct C3 convertase (C3bBb). Subsequently, C3 and C5–9 are sequentially involved in the later steps of all three pathways with the generation of C3a and C5a [15]. Accordingly, the complement was mainly activated through the alternative pathway in our HSP patients. In addition to the alternative pathway, Hisano S et al found the activation of lectin pathway in some HSP nephritis patients with advanced glomerular injury and prolonged urinary abnormalities. [25]. Only 6 of 30 patients in this study had renal involvement. Besides, the disease severity was mild in most of them that indicated the less possibility of lectin pathway activation in these patients, and may explain why the serum levels of C4 were not changed between acute and convalescent stages and the plasma levels of C4a were not increased in those patients at the acute stage.

C3a and C5a are small protein fragments released from the cleavage of C3 and C5. Their plasma levels in HSP patients were high at the acute stage and significantly declined when HSP was in remission. Since they are powerful inflammatory mediators, it is of interest to clarify the effects of C3a and C5a on endothelium of cutaneous small vessels (i.e. HMVEC-d) that are majorly affected in HSP. First, the HMVEC-d were shown to constitutively express the receptors of C3a (C3aR) and C5a (CD88) without any stimulation. We further wanted to evaluate the expression levels of above receptors on inflamed endothelium of HSP. However, it was very difficult to obtain the skin specimens from children with HSP. Therefore, we conducted an in vitro experiment to analyze HMVEC-d incubated with plasma of patients with acute HSP or controls instead of EC derived from patients. Such assay revealed that acute HSP-derived plasma did not interfere with the expression of C3aR and CD88 on HMVEC-d.

Endothelium is a major component of blood vessels and plays an important role in the process of inflammation including vasculitis [26]. Generally, EC are a target for a variety of exogenous and endogenous stimuli, which can induce EC to produce various inflammatory mediators [27]. In the current study, C3a at a higher concentration enhanced the production of IL-8; and C5a in a dose-dependent manner up-regulated the production of both IL-8 and MCP-1 by HMVEC-d. IL-8 is a potent chemoattractant that induces the migration of PMN to the sites of inflammation. Although MCP-1 does not directly recruit PMN, it activates mast cells to generate lipid mediators including leukotriene B4 and platelet activating factor that mediate firm adherence and transmigration of neutrophils [28,29]. The recruitment of PMN into inflamed tissue needs not only chemotactic factors but also adhesion molecules on both PMN and EC. E-selectin and ICAM-1, two most important adhesion molecules expressed on EC, are responsible for cell rolling and tight binding to endothelium, respectively. Human umbilical venous endothelial cells (HUVEC) were commonly used to evaluate the effects of C3a and C5a on the endothelial expression of adhesion molecules. Some previous studies showed that C3a and/or C5a did not alter the expression of E-selectin and ICAM-1 on HUVEC [30,31], while Albrecht EA et al found that C5a could enhance progressively the above two adhesion molecules in HUVEC by microchip gene arrays and reverse transcriptase polymerase chain reaction rather than flow cytometry [32]. Moreover, another human EC derived from choroidal capillaries have been shown to response to C5a by increasing ICAM-1 mRNA and protein [33]. Our in vitro test then revealed for the first time that those HMVEC-d stimulated by C5a resulted in a dose-dependent increase in the expression of both E-selectin and ICAM-1.

The complement proteins are primarily generated by hepatocytes, but other tissues and cells including EC have also been shown to synthesize and secret these proteins [34]. What complement proteins that have been reported to be expressed by human EC, especially HUVEC included C1s, C1 inhibitor, C3, C5–9, and factor B, H, and I [35,36,37]. We herein found both C3 and C5 could be detected by ELISA in the serum-free culture supernatant of HMVEC-d which were pre-treated by human plasma. Importantly, HSP plasma-stimulated HMVEC-d produced more C3 and C5 than those cells stimulated by plasma of healthy controls. As a result, such reaction may locally provide additional complement proteins to participate in the process of vasculitis. More studies are thereafter needed to identify the potential factors for the enhancement of endothelial C3 and C5 secretion in acute HSP plasma.

**Fig 7:**
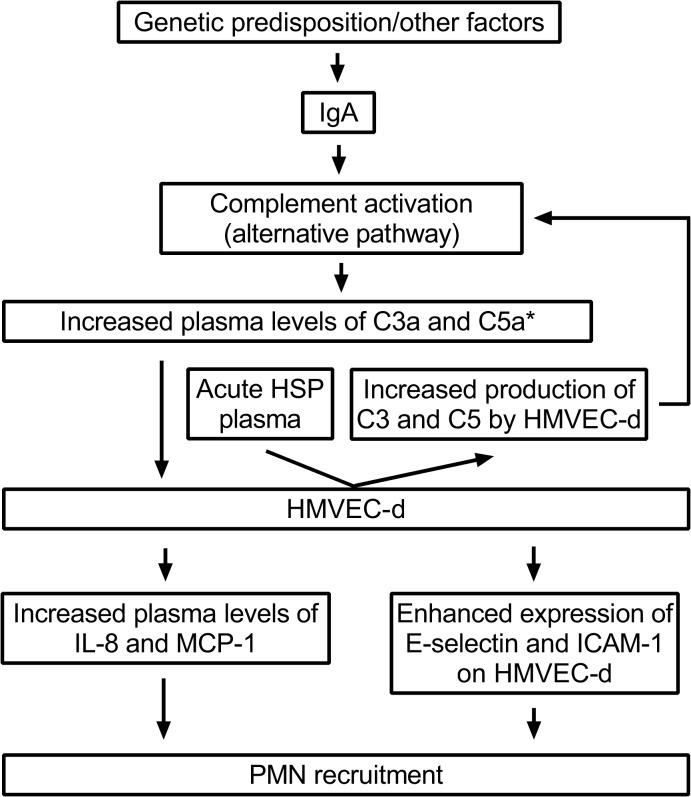
The hypothetic model of complement activation in the pathogenesis of childhood HSP. * The expression of IL-8, MCP-1, E-selectin, and ICAM-1 by HMVEC-d was majorly enhanced by C5a.

In summary, our current study indicated the activation of alternative pathway in HSP based on the elevated plasma levels of C3a, C5a, and Bb, but not C4a in HSP patients at the acute stage. Acute HSP-derived plasma although did not affect the expression of C3aR and CD88 on EC, it possessed the ability to enhance the local production of C3 and C5 by HMVEC-d. Furthermore, bioactive complement fragment C5a up-regulated the endothelial expression of IL-8, MCP-1, E-selectin, and ICAM-1 that are critical molecules for PMN accumulation (Fig. 7). Together, these data seem to support the hypothesis that circulating complement proteins are involved in the pathogenesis of childhood HSP, and also provide new information for a better understanding of HSP.
